# Year-Long Assessment of Soil Nematode Diversity and Root Inhibition-Indicator Nematode Genera in Rice Fields

**DOI:** 10.3390/biology11111572

**Published:** 2022-10-26

**Authors:** Rawhat Un Nisa, Anees Un Nisa, Ali Ahmed Hroobi, Ali Asghar Shah, Aadil Yousuf Tantray

**Affiliations:** 1 Nematode Biodiversity & Genomics Research Lab, BGSB University, Rajouri 185234, India; 2 Mycology & Plant Pathology Lab, Department of Botany, University of Kashmir, Srinagar 190006, India; 3 Department of Biology, College of Sciences in Almandaq, Al-Baha University, Al-Baha 65779-7738, Saudi Arabia; 4 Department of Botany, Aligarh Muslim University, Aligarh 202002, India; 5 School of Biological Sciences, University of Aberdeen, Aberdeen AB243UU, UK

**Keywords:** abundance, free-living nematodes, paddy, plant-parasitic nematodes, prominence value, rice root inhibition, seasons, soil nematode community

## Abstract

**Simple Summary:**

Nematodes are the soil microbes that function for nutrient regulation and biological degradation. Nematode diversity changes during different seasons of a year due to changes in ecological factors. Soil characteristics change significantly due to lowland rice cultivation. This research investigated soil nematode diversity and seasonal changes in rice fields during three months of the year. Soil nematode abundance was different during the three seasons, and plant-parasite nematodes were more abundant during summer than in spring or winter. Soil characteristics, such as soil moisture, carbon content, and nitrogen content, were more common during the summer season than in the spring and winter seasons, while soil pH was low in the summer season. The plant-parasitic nematodes showed a stronger correlation with the soil characteristics during the summer season than in the spring and winter seasons. In addition, the abundance of some free-living nematode genera functioned as ecological indicators. The community and diversity indices of this study will help farmers and microbiologists in nematode management in crop fields.

**Abstract:**

Soil nematodes contribute to nutrient cycling. This year-long study aimed to investigate the changes in the diversity of soil nematodes during the spring, summer, and winter seasons in rice fields at 24 sites and to determine the indicator nematode genera that inhibit the roots of rice plants. A total of 216 soil samples were collected during three seasons, and the collection of 72 root samples was carried out during rice cropping. Forty-four soil nematode genera were identified. They exhibited significant changers in their abundance, which were dependent on the seasons and on soil characteristics. In particular, the abundance of plant-parasitic nematodes (PPNs) and free-living soil nematodes was 49% and 15% higher during the summer than during the spring and winter seasons, respectively. Soil characteristics, such as soil nitrogen (N) contents, carbon (C) contents, and soil moisture were significantly higher during the summer than in the spring and winter seasons, but soil pH was significantly lower during the summer than in the spring and winter seasons. Moreover, *Hirschmanniella,* *Meloidogyne,* and *Heterodera* emerged as good indicators for rice root inhibition, corroborating the frequency, density, and prominence value of PPNs of the sampled soil and rice roots. This study also indicated that free-living nematode genera, such as *Rhabdolaimus, Diplogaster,* and *Rhabditis*, might function as ecological indicators for soil health.

## 1. Introduction

More than half of the world’s population depends on rice as the primary source of food, most of which (>90%) is cultivated and consumed in Asia [1]. Nematodes parasitize most crop plants and cause over USD 150 billion of crop loss per year globally [2,3]. In most cultivated crops, plant-parasitic nematodes (PPNs) live as parasites on roots, shoots, or both. Previous studies have reported that more than 35 genera and 130 species of PPNs are associated with rice plants [4]. The economically important nematodes that cause significant crop losses include *Aphelenchoides besseyi* (white tip nematode), *Meloidogyne graminicola* (rice root-knot), *Pratylenchus zeae* (root lesion), *Hirschmanniella oryzae* (rice root), and *Ditylenchus angustus* (rice stem) [5,6]. *Meloidogyne incognita* has a wide range of pathogenicity and significantly affects crop plants grown under different conditions [7]. In addition to *Hirschmanniella oryzae*., *M. graminicola* causes severe loss to the rice fields [5,8].

Nematodes are also beneficial for nutrient recycling and the establishment of sustainable ecologies [9,10,11]. Nematodes are involved in decomposition at the soil surface after crop harvesting [12]. Additionally, nematodes act as regulating factors at various trophic levels of the soil food web and help evaluate the composition and structural development in the food web and ecosystem maturity [13,14]. Major physiochemical soil parameters, including pH, moisture content, temperature, and composition of the soil, change with seasonal changes during the year. Increased water content increases soil pH in the broad spectrum but decreases soil pH with increased salt concentrations [15]. Omnivores and predatory nematodes are highly sensitive to environmental disturbances, resulting in a higher number of nematodes in natural land than in disturbed agricultural land [16]. The addition of organic and inorganic fertilizers brings changes in soil parameters such as texture, porosity, and pH, which result in an increased diversity of free-living nematodes [17,18]. Generally, plant-parasitic nematodes prefer soil with slightly acidic pH to flourish their populations [19,20]. A 1992 study found that acidic paddy soils with high organic content increased pH after three days of water flooding, which slightly decreased and stabilized after half a month [15].

The primary challenge is checking the nematode populations in rice fields eco-sustainably, without affecting crop production and soil mineralization. Crop rotation and cultural practices (flooding and fallowing) have decreased nematode populations and increased yield [21]. The assimilation of harvest remains in paddy soils reduced penetration resistance, bulk density, and soil compaction under crop rotation systems [22,23,24,25]. However, there is a need for the biological management of nematode populations in rice fields to conserve production for the increasing global population. Among bio-indicators, soil nematodes are best for checking soil quality and ecosystem functioning, due to their sensitive community composition, nutrient enrichment, and management changes [13,26]. In addition, the community composition of nematodes changes considerably faster than plant communities and nematodes are known as fast colonizers [13,27]. The fast changes in nematode community composition help to determine soil quality and ecological disturbances. The diversity indices of nematodes and their community structure help in determining specific ecological management and food chain strategies [11,28,29,30]. Recently, nematodes were found to be helpful in enhancing the innate immune responses of plants against pathogens via secreting ascarosides [31,32]. Despite the facts highlighted above, the literature exploring the successional maturity or trophic diversity of nematode communities for sustainable management in crop fields, which may ensure food security, is meager.

Given the above, this study hypothesizes that the abundance and diversity of nematodes of topsoil in rice fields change with soil characteristics during different seasons of the year. A large-scale experiment was designed for the following purposes: (i) to determine the abundance, frequency, density, and diversity of nematodes in three seasons of the year—spring, summer, and winter; and (ii) to determine the indicator genera among nematodes for soil health and root-inhibition of rice plants.

## 2. Materials and Methods

### 2.1. Study Sites and Sampling Methods

**Figure 1:**
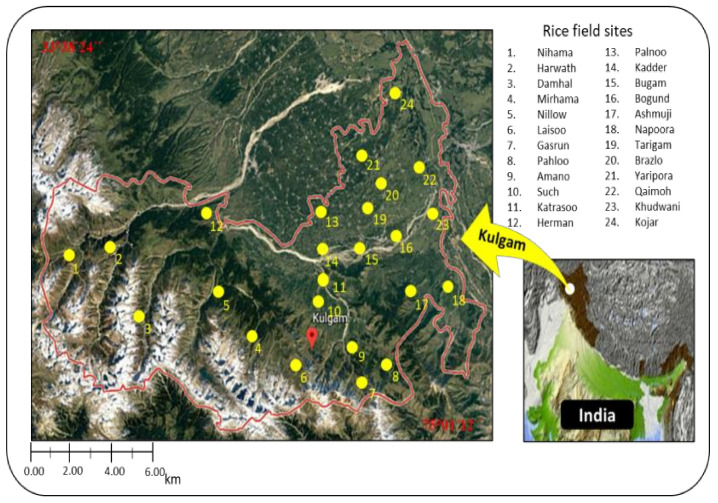
The map of 24 sampling sites of the rice fields of district Kulgam of Jammu and Kashmir, India. Each site is marked with a yellow dot and number on the map; the distance between the sampling sites ranged from 2.10 km (minimum) to 15.40 km (maximum).

The soil and rice root samplings were from the rice fields of district Kulgam (known as the “rice bowl of Kashmir”), a region of Jammu and Kashmir, India. Twenty-four sampling sites of the rice fields were selected within the geographical coordinates of latitude 33°38′24′′ N and longitude 75°01′12′′ E (Figure 1). These selected sites were used for rice crops for many years. The sites were selected based on rice planting times, irrigation systems, and management of fertilization by nitrogen (N), phosphorus (P), and potassium (K) (NPK) (N = 120, P = 40, and K = 40 kg hac ^−1^). A total of 216 independent soil samples were collected during three seasons of the year: spring, summer, and winter. The soil sampling in the spring season was carried out before rice plantation, on 12 April 2018 to 14 April 2018; the summer sampling was carried out when the rice plants were at the maximum tiller stage, on 20 July 2018 to 22 July 2018; the winter sampling was carried out after the rice crop was harvested, on 27 November 2018 to 29 November 2018. Of the soil samples collected, 72 were from each season. At each of 24 selected sites, three random soil samples were collected during each sampling time. For each sample, 500 g of soil were taken, with the help of a soil probe, from 5 cm to 10 cm below ground surface and kept separately in properly labeled plastic bags. The collected soil samples were stored at 4 °C during transfer to the lab for analysis, to maintain moisture.

The rice root samples were collected during the summer sampling from the same 24 selected sites of the rice fields. A total of 72 independent rice root samples were collected from the 24 sites, and at each site, three random root samples were obtained. Fifty grams of the roots were collected from several plants and kept in labeled plastic bags for each sample.

### 2.2. Measurement of Soil pH and Moisture Content

A sub-sample of 20 g of soil from each sample was dissolved in 100 mL of distilled water separately and stirred with a glass rod till the formation of uniform soil suspension. An automatic pH meter was used to determine the pH of the soil suspension. The gravimetric method was employed to determine the soil moisture content. A sub-sample of 10 g of soil was used to measure fresh weight; then, the soil samples were dried in an oven for 24 h at 105 °C before measuring dry weight. The soil moisture contents are represented in percentages using a formula: fresh weight of the sample—the dry weight of the sample × 100/dry weight of the sample.

### 2.3. Determination of Soil Nitrogen and Carbon Content

A CHNS analyzer (EuroVector 3000, Pavia, Lombardy, Italy) measured the soil N and C content based a simultaneous determination by a gas chromatography (GC) system. The instrument was calibrated, and a standard curve was prepared by using certified standards. A sub-sample of 2 g of soil from each sample was oven-dried at 105 °C for 24 h, then finely powdered with the help of a ball mill machine. After that, 10–12 mg of powdered soil was used for analysis, which was completed within 10 min after loading to a combustion reactor [33].

### 2.4. Nematode Extraction from the Soil Samples

For nematode extraction, a sub-sample of 200 g of soil was taken from each sample, mixed well to obtain a uniform distribution of nematodes, and mixed thoroughly with water to obtain soil suspension. The soil suspension was passed through a coarse sieve to remove debris and lumps. The suspension was also passed onto the fine sieve to obtain minor soil particles. Finally, 50 mL volume of soil suspension was used for nematode extraction. Nematodes were isolated sequentially using modified Cobb’s sieving and decantation (with mesh sieves of 833, 74, and 38 µm apertures) and Baermann’s funnel technique [34].

### 2.5. Nematode Extraction from the Root Samples

The nematodes of the rice root samples were extracted by a maceration sieving method [35]. The root samples (50 g) were washed five times, sheared into 2 cm pieces, then air-dried. The root samples were softened for 10 s, crushed in a blender, then transferred to Baermann’s funnel. The succeeding steps were similar to those of nematode extraction from the soil samples.

### 2.6. Identification of Nematode Genera

Glass slides were prepared for the identification of nematodes. Using an Olympus microscope, BX51 nematodes were identified up to the genus level, based on the morphological characters of nematode genera at 100× and 40× magnifications. Trophic groups were allocated according to Yeates [36] and arranged in colonizer-persister (c-p) groups according to Bongers [27]. The counting of nematodes was performed using a Syracuse counting dish. Identification of nematodes up to a generic level was carried out on the basis of appropriate literature [37,38,39]. Hunt [40] taxonomic keys for plant-feeding dorylaimids (Trichodorids and Longidorids) and Aphelenchids were also used.

### 2.7. Nematode Community and Diversity Indices

The relationship between nematodes and rice fields was determined by community analysis. Nine nematode indices were calculated, showing different aspects of the community. The Shannon–Wiener index (H’) and the Simpson index (D) were calculated, which indicated the nematode species diversity in a community [41,42]. Both the Shannon–Wiener index and the Simpson index accounted for the abundance and evenness of the species present. The species evenness was illustrated by Pielou’s evenness index (J’), which is closely related to species dominance [43]. Species richness was measured by the Margalef index (MgI) and was calculated as follows: *MgI =* (*G−1)/ln (n)*, where *G* is the total genera number and *n* is the total number of individuals [44].

Environmental disturbances in the soil were signified by the maturity index (MI) [45,46], based on the relative contribution of nematodes of different c-p values. The MI considered all nematode groups (c-p 1 to 5), and the high MI revealed stable soil conditions. The functional structure of the community was referred to by the Wasilewska index (WI), the channel Index (CI), the plant-parasitic index (PPI), and food web complexity (FWC). The WI represents the ratio of bacterial feeders (BF) plus fungal feeders (FF) to plant-parasites (PP) as WI = (BF + FF)/PP [47]. The CI represents the fungal participation in decomposition channels of soil food webs. The higher value signifies dominated fungal feeding decomposition, whereas the low values indicate a dominated bacterial decomposition pathway [14]. The PPI is similar to MI, but only for plant-parasitic nematodes, i.e., 1/*N* (*c* − *p*)*i* × *ni*, where (*c* − *p*)*i* is the c-p value for plant-parasitic nematodes and *ni* is the total number of individuals of a plant-feeding nematode *i* [48]. The FWC is the ratio between predatory nematodes (PR) and plant-feeding nematodes. It represents the top-down control in which predatory nematodes control herbivores, i.e., plant-feeding nematodes (PR/PF) [49]. The functional indices were calculated using the online program “NINJA: An automated calculation system for nematode-based biological monitoring” [50], https://sieriebriennikov.shinyapps.io/ninja/) (accessed on 5 June 2019).

### 2.8. Statistical Analysis

The datasets were analyzed by a one-way ANOVA model where pH, moisture, nitrogen content of the soil, carbon content of the soil, and soil nematode measures function as responses and seasons function as factors. The absolute frequency (AF), relative frequency (RF), mean density (MD), and relative density (RD) were calculated from the mean data of nematode soil samples. The N denotes the number of samples in which the genus was present, and the AF denotes the frequency of the genus by the total number of soil/root samples. MD is the ratio between the number of nematode specimens counted in all samples and the total number of samples collected. RD represents the percentage of the mean density of the genus × 100/sum of the mean density of all nematode genera. The prominence value (PV), which represents the dominant genus, was calculated as PV = population density √relative frequency, and the relative prominence value (RPV) was calculated as the ratio between the PV of the nematode genus and the sum of the PVs of all nematode genera ×100. The PV and RPV indicate the dominant genus and a relationship of population density and frequency of the identified nematode genera. Analysis of variance was performed by Minitab 19.0, and Duncan’s multiple range test was applied separately for each season. A principal component analysis (PCA) was used on the nematode-community data for the three-season samplings, with explanatory variables of soil pH and moisture. Correlation between nematodes found in root and soil samples was performed to determine indicator plant-parasitic genera.

## 3. Results

The variation of soil factors (pH, moisture, nitrogen content, and carbon content) and nematode indices (density, frequency, and abundance of soil nematodes) were significant between the three seasons of the year in the rice fields (Table 1). The highest variation was depicted by soil pH (66.12%) and moisture (58.23%), followed by soil nitrogen content (48.34%). Among the soil nematodes that were measured, abundance showed the highest variation of 55.25% and frequency showed the lowest variation of 44.61%. In addition, the root nematode frequency depicted significant interaction with the soil nematode frequency of the three seasons in the rice fields.

### 3.1. Soil pH, Moisture, Nitrogen, and Carbon Content in Different Seasons

**Figure 2:**
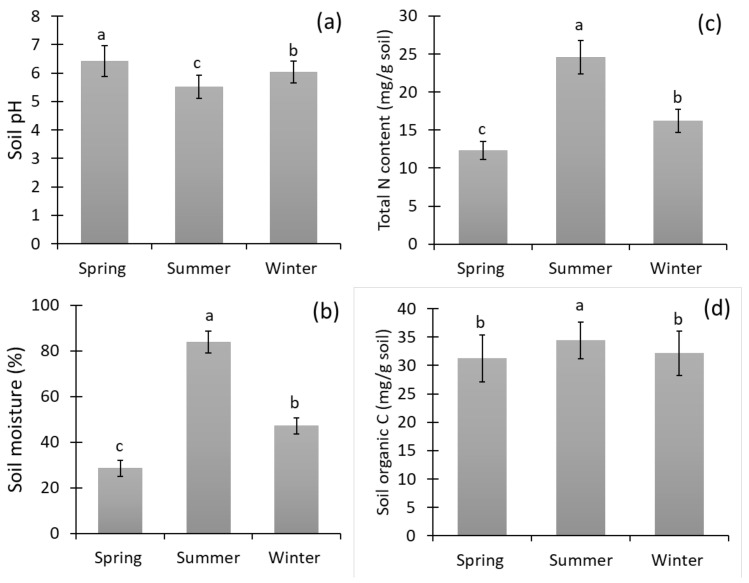
The soil pH (**a**), moisture content (**b**), total nitrogen content (**c**), and total carbon content (**d**) of the soil samples of the rice fields during the three seasons of the year. Bars represent the mean of 72 soil samples (± SD) and different letters on the bars show the significance at *p* ≤ 0.05 among the different seasons.

There was a significant change in soil pH, moisture content, total nitrogen content, and soil carbon content between the three seasons of the year in the rice fields (Figure 2). The highest soil pH (6.80) was observed in spring, followed by the winter and summer seasons, whereas the highest soil moisture content was during summer (87.50%), followed by the winter (50.10%) and spring (31.20%) seasons. The soil nitrogen content (24.21 mg g^−1^ soil) and carbon content (34.56 mg g^−1^ soil) were highest in summer, compared with the winter and spring seasons.

### 3.2. Abundance of Soil Nematodes in Different Seasons

**Figure 3:**
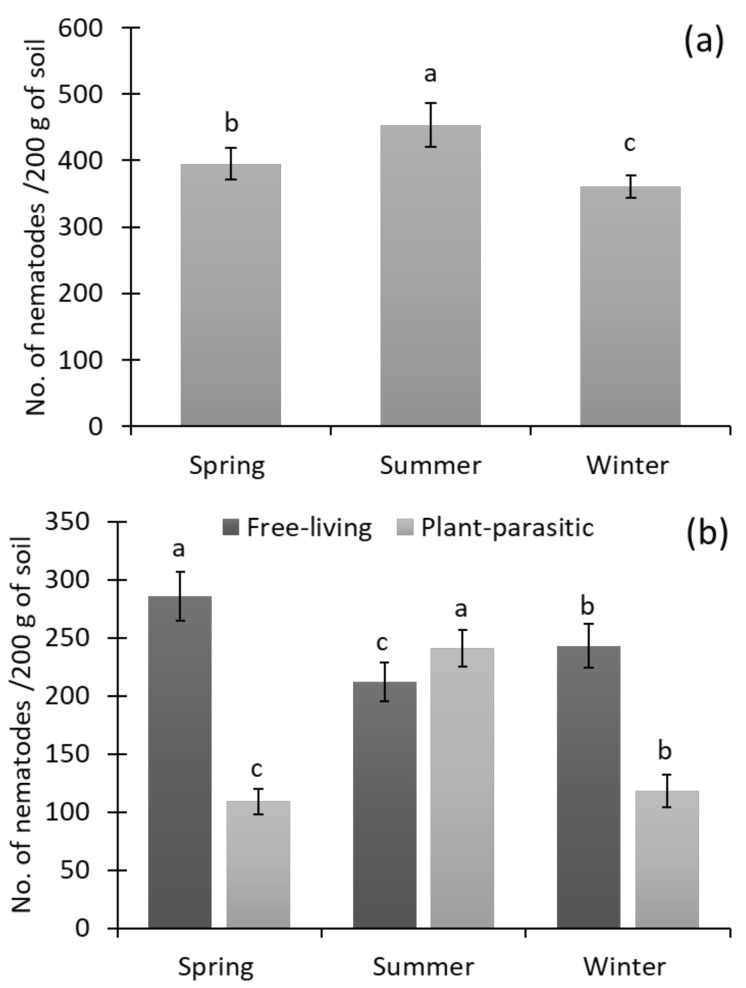
The soil nematode abundance (**a**), and contribution of free-living and plant-parasitic nematode abundance (**b**) in the rice fields at the three different seasons of the year. Bars represent the mean of 72 soil samples (± SD) and different letters on the bars show the significance at *p* ≤ 0.05 among the different seasons.

A significant change was observed in nematode abundance during the three seasons of the year (Figure 3). The nematode abundance was highest in summer, followed by spring and winter. The contribution of PPNs was observed to be highest in the summer season and lowest in the spring season. In contrast, the highest contribution of free-living nematodes was found in the spring and the lowest was found in the summer season in the rice fields.

### 3.3. Trophic Nematode Structure in Different Seasons

**Figure 4:**
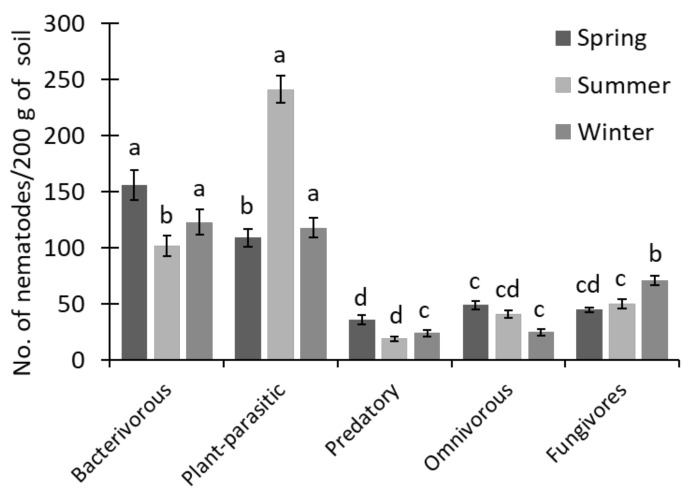
The trophic structure of the soil nematodes of the rice fields during the three different seasons of the year. Bars represent the mean of 72 soil samples (± SD) and different letters on the bars show the significance at *p* ≤ 0.05 among the different nematode groups at each season, separately.

A diversified nematode population was found during the different seasons of the year (Figure 4). Bacterivorous nematodes and PPNs dominated over other nematode groups during the spring and summer seasons, respectively. Nevertheless, they were equally dominant in the rice fields during the winter season. A minor abundance of nematodes was observed in the predatory group during each of the year’s three seasons.

### 3.4. Variation of Soil Nematode Frequency and Density in Different Seasons

**Figure 5:**
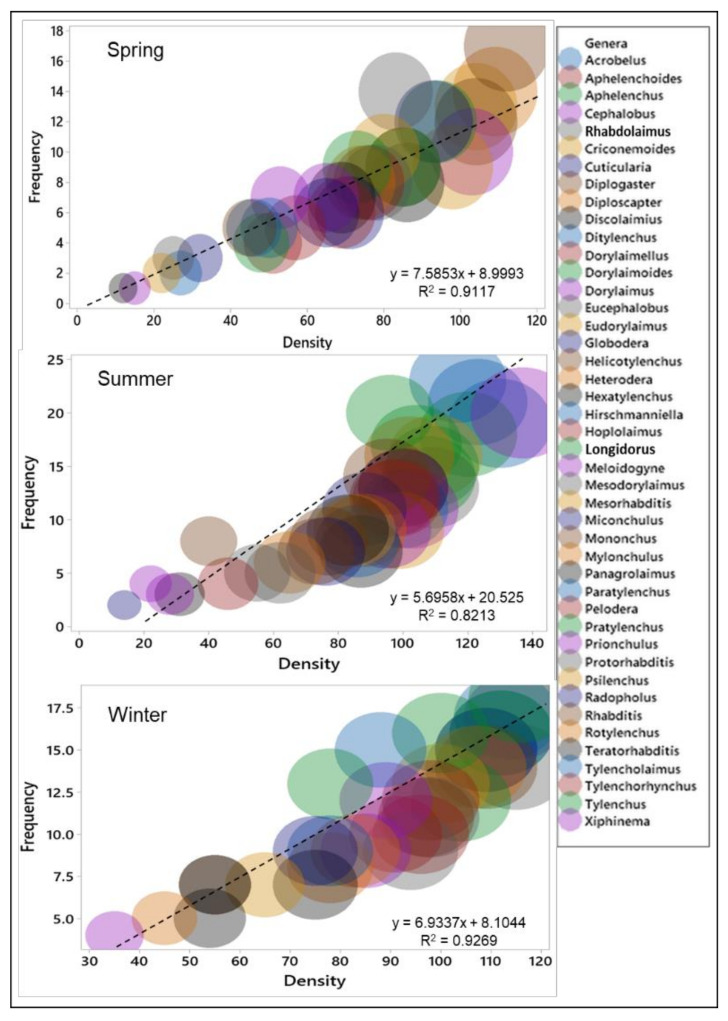
The frequency and density relation of soil nematodes during the spring, summer, and winter seasons of the year in the rice fields. Linear trendlines were obtained from mean data of the frequency and the density of the soil nematode genera; R^2^ represents the correlation strength in the three seasons.

The frequency and density of nematode genera varied frequently in three seasons of the year (Figure 5). During spring, the genus *Diplogaster* had the highest frequency (17) and density (112). During the summer season, the genus *Hirschmanniella* had the highest frequency (23) and the genus *Meloidogyne* had the highest density (130). During the winter season, the genus *Dorylaimellus* had a higher frequency (18) and density (116) among the 44 nematode genera found in the soil samples. Similar trends were observed in other nematode diversity measures, such as absolute frequency, relative frequency, mean density, and relative density (see the Supplementary Materials). A correlation between the density and the frequency of the nematode genera of soil samples changed with the seasons in the rice fields. During the winter season, the density and frequency of the nematode genera predicted a strong correlation (R^2^ > 0.9117; *p* = 0.021), which decreased in spring, but little correlation (R^2^ = 0.8213; *p* = 0.035) was observed during the summer season in the fields.

### 3.5. Shift of PPNs during Different Seasons in the Rice Fields

**Figure 6:**
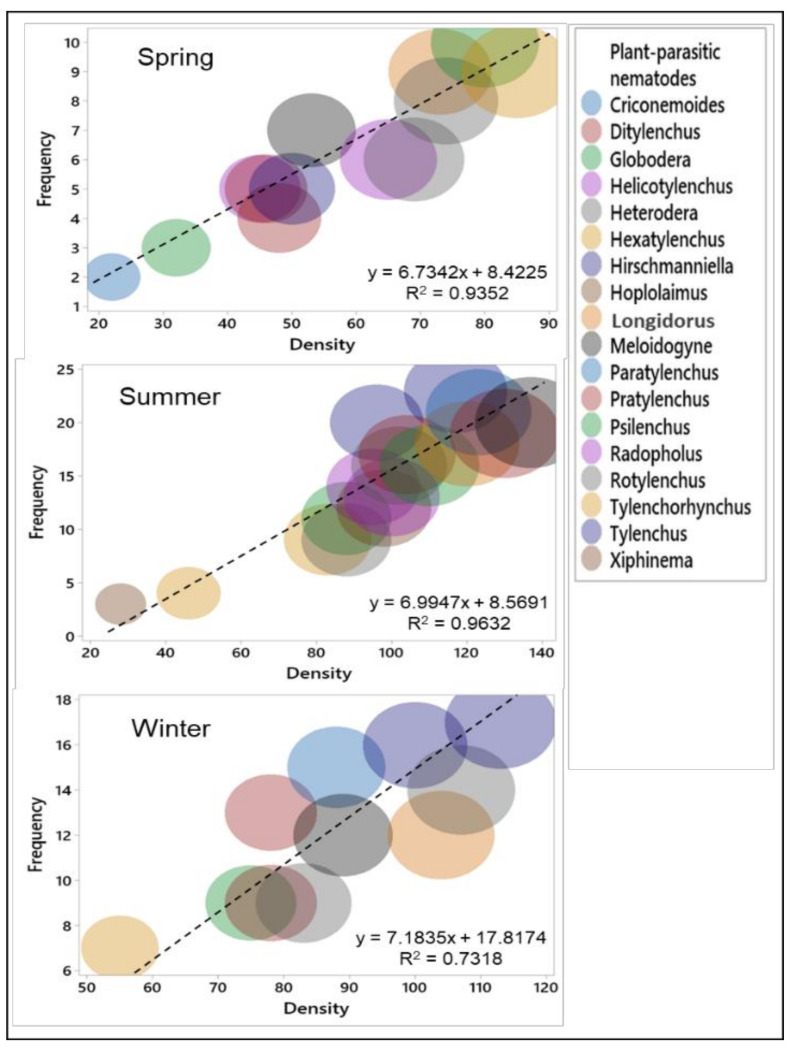
The changes in plant-parasitic nematode diversity of the soil during the spring, summer, and winter seasons in the rice fields. Linear trendlines were obtained from mean data of the frequency and the density of the soil PPN genera; R^2^ represents the correlation strength in the three seasons.

The PPNs showed dynamic variation with the seasonal changes of the year in the rice fields (Figure 6). Genera such as *Longidorus, Meloidogyne, Hirschmanniella, Tylenchus*, and *Paratylenchus* were persistent during the three seasons of the year. These genera displayed more than a two-fold increase in frequency and density during the summer season, and more than a one-fold increase during the winter season, compared with the spring season. PPN genera such as *Rotylenchus, Hoplolaimus, Hexatylenchus,* and *Xiphinema* appeared only in the summer season in the rice fields. A correlation between the density and the frequency of PPN was strong during the summer season (R^2^ = 0.9632) and weak during the winter season (R^2^ = 0.7318).

### 3.6. PV and RPV of Nematode Genera in the Rice Fields

The soil nematode genera prominence and relative prominence were diverse during the three seasons of the year in the rice fields (Table 2). The highest prominence value (PV = 459) was observed during spring in the genus *Rhabdolaimus*, while the least PV was found in the genus *Clarkus* (PV = 11.95). Among the PPNs, the genus *Meloidogyne* had the maximum PV (351.5), followed by the genus *Ditylenchus* (PV = 325.5), while the least PV was noted in the genus *Xiphinema* (PV = 27.8).

During the spring season, the genus *Rhabdolaimus* had the highest relative prominence value (RPV = 6.4), followed by the genus *Diploscapter* (RPV = 5.7), while the least RPV was found in the genera *Acrobelus* and *Protorhabditis* (RPV = 0.2). During the summer season, the highest RPV (87.8) was found in the genus *Meloidogyne*, followed by the genus *Ditylenchus* (RPV = 81.2), and the lowest (RPV = 2.8) was found in the genus *Miconchulus*. The genus *Dorylaimoides* displayed the highest relative prominence value (RPV = 82.5) during the winter season, while the lowest (PRV = 3.4) was found in the genus *Longidorus.*

### 3.7. Principal Component Analysis of Soil Nematodes

**Figure 7:**
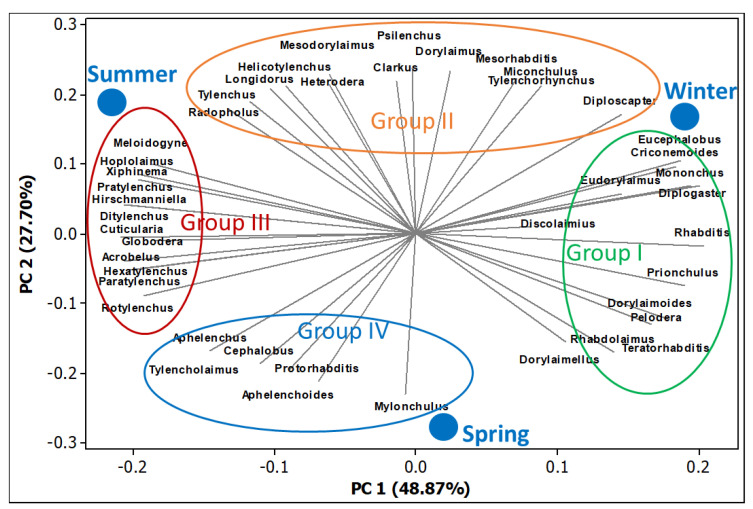
Principal component analysis (PCA) of soil nematode genera during the three seasons: spring, summer, and winter. The PCA shows the season’s position according to nematode diversity and the contribution of the nematode genera in the four groups.

The nematode-community data was used for the PCA, which determined the favorable position of nematode diversity during the spring followed by the summer and winter seasons of the year (Figure 7). Four groups of nematodes were determined in the PCA plot, based on PC 1 and PC 2 contributions. Group-I mainly consisted of bacterivores; group II mainly consisted of omnivores, bacterivores, and PPNs; group III mainly consisted of PPNs; and group IV mainly consisted of predatory and fungivores. According to the position of the groups, PPNs had a strong correlation with bacterivores during the change of seasons durng the year. At the same time, the predatory and the fungivores had a strong correlation with the omnivores and some genera of the bacterivores and PPNs during the different seasons of the year.

### 3.8. Nematode Genera Inhibited Rice Roots

**Figure 8:**
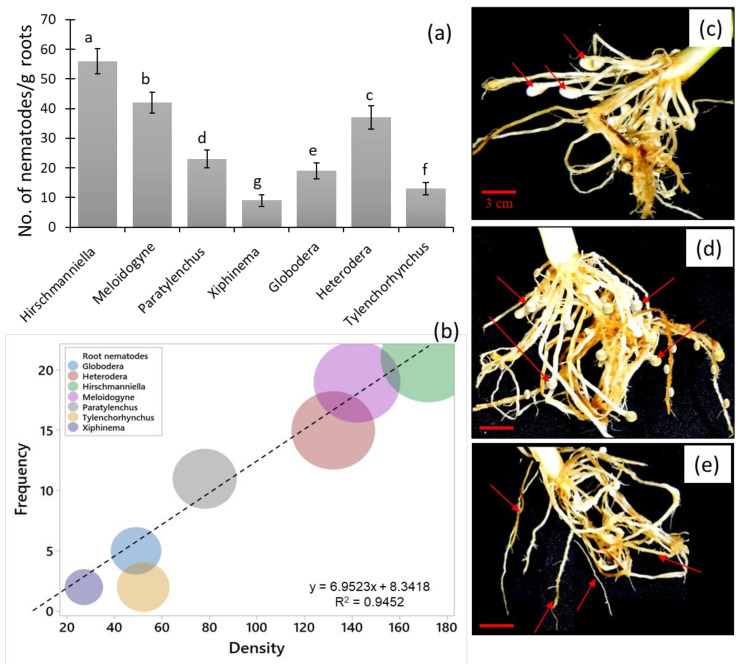
The nematode genera are found in the rice root samples during crop plantation (summer season) in the rice fields. (**a**) The abundance of root-nematode genera, where bars represent the mean of 72 rice root samples (± SD) and different letters on the bars show the significance at *p* ≤ 0.05 among the different nematode genera; (**b**) frequency and density relation of root nematodes in the rice fields; (**c**) root-knot infection by nematodes; (**d**) cyst formation by root nematodes; and (**e**) lesion formation by root nematodes.

Seven parasitic nematode genera were found in the root samples of the rice plants (Figure 8). The nematode genera *Hirschmanniella* showed the highest abundance in root samples, followed by *Meloidogyne*; the abundant minor genus was *Xiphinema*. Three root-nematode genera with high frequency and density, such as *Hirschmanniella* (F = 21), *Meloidogyne* (F = 19), and *Heterodera* (F = 15), were indicators of the nematode community. The effects caused by the dominant root nematodes on the rice root were root-knots, cysts, and lesion formations (Figure 8c–e). Among the root nematodes, *Hirschmanniella* had the highest (4.87) relative frequency, followed by *Meloidogyne* (4.41) and *Heterodera* (3.21), while *Xiphinema* (0.58) had the lowest. Similar trends were observed in absolute frequency, mean density, relative density, prominence value, and relative prominence value of the root-nematode genera in the rice root samples (Table 3).

### 3.9. Community and Diversity Indices of Soil Nematodes

The community and diversity indices of the soil nematodes in the spring, summer, and winter seasons explained the soil structure and stability of the rice fields (Table 4). The highest H’ was observed in summer (3.60) and the lowest in winter (3.40). The J’ (1.00), MgI (6.21), and CI (9.10) mainly dominated in the summer season in the rice fields. The highest WI (1.50) was observed during the winter season in the rice fields. In addition, the D (0.97), FWC (0.60), and MI (2.40) of the soil nematodes were most significant during the spring season in the rice fields. The highest (2.97) PPI of the soil nematodes was found in summer, while the lowest (2.47) PPI was found in winter.

## 4. Discussion 

In the present study, nematode diversity was examined in Indian rice fields during the spring, summer, and winter seasons. It was found that the bacterivorous nematodes dominated at higher pH. In contrast, the plant-parasitic nematodes dominated in acidic pH of the soil and decreased the bacterial feeders during all three seasons in the rice fields (Figure 2 and Figure 4). However, omnivores were not much affected by the change of soil pH during the seasons. A previous study found that low pH decreases bacterivorous nematodes but increases fungivorous nematodes [51]. Factors such as soil pH and soil moisture significantly impact the nematode community, and their changes result in a change of the nematode community structure [13,52,53]. The habitat of nematodes [54] and their density, frequency, and diversity change with changing edaphic and ecological factors [55,56]. The pH change in the rice fields and nematode abundance may occur due to inorganic fertilizers [57,58,59,60]. In addition, soil moisture plays an essential role in the distribution of nematodes. The PPNs were highest with higher soil moisture content, followed by predatory and bacterivorous nematodes. Thus, soil moisture is a regulating factor for nematode abundance [61]. In some instances, the low soil moisture causes the reproduction rate in nematodes to decline [62], as it functions as a medium during interchange [63]. Based on the above observations and statements, nematodes are indicators for community analysis, soil health, and water conditions for soil microbes in changing environmental habitats [13,30,64].

During three seasons, the nematode dominance varied, and free-living nematodes were dominant in the spring and winter seasons; however, PPNs were dominant in the rice fields during the summer season. Nematode frequency and density were lower during summer (r^2^ = 0.8213) than in the spring (r^2^ = 0.9117) and winter (r^2^ = 0.9269) seasons in the soil samples (Figure 5). The decrease in free-living nematode populations during summer may be due to the low adaptation of these nematodes under flood irrigation conditions (anaerobic conditions) in the rice fields. Among the five trophic groups of 44 identified soil nematode genera, PPNs constituted the highest genera in terms of abundance in the rice fields. These results coincide with those of previous studies [65]. The higher population of plant-parasitic nematodes during the summer rice crop may be due to the growing crop, manures, and fertilizers [58,59]. Among the PPN genera, *Hirschmanniella,* *Meloidogyne, Heterodera,* *Tylenchus, Paratylenchus,* and *Longidorus* had high frequency and density during all three seasons of the year. However, regarding the frequency of the genera, *Hirschmanniella,* *Meloidogyne,* and *Heterodera* increased more than two-fold during the summer rice crop than the spring season (with no crop) (Figure 6). The dominance of these three nematode genera was previously reported in rice fields worldwide [66,67]. Among the free-living nematode genera, *Diplogaster, Rhabditis, Mesodorylaimus, Dorylaimoides,* and *Aphelenchus* may function as indicators for community structure and soil stability in rice fields, due to their persistence during all three seasons (Table 2).

The three nematode infections (root-knots, cysts, and lesion formations) associated with seven PPN genera were found in the roots of rice plants in this experiment. Root-knot infections in rice was mostly caused by *Meloidogyne graminicola*; cysts were caused by the *Heterodera* species; and lesion formations were caused by the *Pratylenchus* species. Of seven identified root-nematode genera, *Hirschmanniella,* *Meloidogyne,* and *Heterodera* dominate rice roots. The frequency of the three root nematode genera approached the frequency of nematodes in the soil during summer; in the spring, it was was < 0.5-fold (Figure 6 and Figure 8). These results suggest that the frequency and density of *Hirschmanniella,* *Meloidogyne,* and *Heterodera* function as the best indicators for the nematode inhibition rate in the rice roots and the position of PPNs in the soil community structure. In previous reports, *Meloidogyne* was the most prevalent and abundant nematode in flooded and rainy lowland areas [68,69,70,71]. It has worldwide dispersion [67] and its spread potential to different topographical places [72] creates an in-time alarm of possible crop destruction in the future. Similarly, *Hirschmanniella* and *Heterodera* have great potential to spread to other ranges of topography, especially in rice fields, and to harm agriculture [6,73].

The H’ was highest during summer (H′ = 3.6), which suggests high diversity. The H’ is used to characterize species diversity in a community and helps find the disturbances of the habitat [74]. Meanwhile, the J′ was also highest during summer, which signifies greater evenness because of resource distribution [43]. Other indices, such as the MgI, PPI, and CI of soil nematodes, were significant in the rice fields during summer. The value of MgI was 6.12 during summer, which explains the high species richness in a community [75], which may be due to fertilizers in the rice fields. The PPI was highest (2.97) in the soil nematodes during summer; this refers to dominance and better adaptation under flood irrigation. The CI was a good indicator of decomposition [58,76]. It was highest (9.1) during summer and lowest during spring; the lower values explain the dominant bacterial pathway in the spring season of the rice fields. The FWC and MI of the soil nematodes were more significant during spring in the rice fields. The decrease of FWC during summer may be due to the abundance of PPNs in the soil. The MI value shows disturbance in the soil [48], and it was found to be lowest (2.2) during the winter season in the rice fields, which is due to the dominance of bacterial feeders. The decreased MI suggests the nematode community’s decreasing structure, because the FWC decreased with increasing N deposition in the soil during summer. The soil mineralization process was indicated by WI and explained the relative balance of positive-to-negative impacts of nematodes on primary productivity [77,78], which was highest (1.50) during the winter season. Due to agricultural practices, immense trophic diversity was mainly associated with an increased frequency of less abundant groups, such as predator groups, fungivorous groups, and omnivore groups [49]. 

Rice cultivation continuously alters the diversity and abundances with different feeding habits, and thus alters the overall complexity and architecture of the detritus food web, which coincides with the findings of Korobushkin [79]. The above-mentioned community and diversity indices of soil nematodes are helpful in investigating soil stability and efficiency, explaining life strategies to exist as colonizer-persisters, and feeding pathways. Some factors regulate nematode diversity indices; for example, the application of manures and fertilizers [59,80].

## 5. Conclusions

The composition and structure of nematodes exhibited apparent diversity in rice fields during three seasons. The pH of the soil was not the same during the three seasons, and higher nematode abundance was observed, at slightly acidic pH, during the summer. The acidic nature of the soil during the summer decreased free-living nematodes and, in turn, increased PPNs and possibly affected the growing crop. A disruption of nematode trophic communities during the summer occurred because of the amplification of the PPNs. *Hirschmanniella,* *Meloidogyne,* and *Heterodera* were identified as the best indicators for rice root inhibition and soil health. These PPN genera could also function as indicators for crop management in rice fields, because they exhibited the same frequency rate in the rice roots and the soil samples during the summer season. In addition, *Diplogaster, Rhabditis,* and *Aphelenchus* were important ecological balancing indicators for soil health, due to their persistence in the soil during all three seasons. The community and diversity indices of this study will assist farmers and microbiologists in nematode management in rice fields.

## Figures and Tables

**Table 1:** One-way ANOVA analysis of soil ecology—pH, moisture, nitrogen content, and carbon content; soil nematode traits—density, frequency, and abundance; and interaction between soil and root nematodes.

Soil Ecology	*p*-Value	*F*-Value	R^2^
Soil Ph	0.006	25.23	66.12%
Soil moisture	0.021	41.89	58.23%
Soil nitrogen content	0.009	38.06	48.34%
Soil organic carbon	0.014	42.31	42.95%
Soil nematode density	0.008	37.81	47.70%
Soil nematode frequency	0.012	31.24	44.61%
Soil nematode abundance	0.000	48.32	55.25%
Interaction of nematode frequency of root samples with soil samples at different seasons			
Root-nematode × soil-nematode (spring)	0.000	27.19	68.73%
Root-nematode × soil-nematode (summer)	0.011	11.41	34.56%
Root-nematode × soil-nematode (winter)	0.002	22.53	57.87%

**Table 2:** The prominence and relative prominence values of the identified soil nematode genera with respective c-p values in the different seasons in the rice fields.

Nematode Genera	Spring Season			Summer Season			Winter Season		
	c-p Value	PV	RPV (%)	c-p Value	PV	RPV (%)	c-p Value	PV	RPV
**Plant-parasitic**									
*Psilenchus*	2	251.98 ± 16.43	3.54 ± 0.56	2	252.46 ± 16.34	63.12 ± 5.12	0	0.00 ± 0.00	0.00 ± 0.00
*Radopholus*	2	158.59 ± 11.32	2.23 ± 0.42	2	206.88 ± 13.19	51.72 ± 4.21	0	0.00 ± 0.00	0.00 ± 0.00
Longidorus *	5	218.13 ± 14.12	3.07 ± 0.51	5	292.12 ± 16.35	73.03 ± 5.69	5	241.83 ± 14.21	3.44 ± 0.55
*Rotylenchus*	0	0.00 ± 0.00	0.00 ± 0.00	1	151.48 ± 12.27	37.87 ± 3.56	1	167.16 ± 12.24	41.79 ± 5.63
*Criconemoides $*	3	30.99 ± 4.21	0.44 ± 0.11	0	0.00 ± 0.00	0.00 ± 0.00	0	0.00 ± 0.00	0.00 ± 0.00
*Ditylenchus*	2	102.45 ± 7.65	1.44 ± 0.21	2	325.13 ± 18.57	81.28 ± 5.78	2	157.09 ± 11.23	39.27 ± 4.82
Meloidogyne *	3	139.67 ± 9.78	1.96 ± 0.25	3	351.54 ± 19.48	87.89 ± 5.88	3	206.97 ± 13.25	51.74 ± 5.76
*Helicotylenchus*	3	100.22 ± 6.89	1.41 ± 0.22	3	203.95 ± 12.23	50.99 ± 4.35	0	0.00 ± 0.00	0.00 ± 0.00
Heterodera *	3	208.47 ± 12.68	2.93 ± 0.30	3	234.10 ± 14.09	58.52 ± 4.43	3	268.76 ± 15.69	67.19 ± 6.21
Hirschmanniella *	3	111.36 ± 7.98	1.57 ± 0.24	3	321.95 ± 18.64	80.49 ± 6.11	3	312.77 ± 18.52	78.19 ± 6.93
*Hoplolaimus*	0	0.00 ± 0.00	0.00 ± 0.00	1	194.79 ± 12.51	48.70 ± 4.68	0	0.00 ± 0.00	0.00 ± 0.00
*Globodera*	3	55.21 ± 5.59	0.78 ± 0.18	3	167.46 ± 12.8	41.87 ± 3.98	3	151.04 ± 11.06	37.76 ± 4.25
Tylenchus *	2	253.99 ± 17.35	3.57 ± 0.63	2	246.34 ± 15.35	61.58 ± 5.24	2	268.52 ± 14.52	67.13 ± 6.22
*Paratylenchus*	0	0.00 ± 0.00	0.00 ± 0.00	1	323.41 ± 18.43	80.85 ± 5.35	1	228.80 ± 12.67	57.20 ± 6.02
*Pratylenchus*	3	95.62 ± 6.11	1.34 ± 0.35	3	246.04 ± 14.65	61.51 ± 5.13	3	188.79 ± 10.45	47.20 ± 5.09
*Hexatylenchus*	0	0.00 ± 0.00	0.00 ± 0.00	2	142.87 ± 12.27	35.72 ± 3.36	2	97.69 ± 8.66	24.42 ± 3.65
*Tylenchorhynchus*	2	168.35 ± 12.25	2.37 ± 0.36	2	52.79 ± 5.62	13.20 ± 1.44	0	0.00 ± 0.00	0.00 ± 0.00
*Xiphinema*	0	251.98 ± 15.97	3.54 ± 0.66	1	27.83 ± 4.15	6.96 ± 0.67	0	0.00 ± 0.00	0.00 ± 0.00
**Bacteriovores**									
*Acrobelus*	0	14.94 ± 2.11	0.21 ± 0.09	2	190.30 ± 12.33	47.57 ± 4.21	2	300.75 ± 17.34	75.18 ± 8.23
Cephalobus *	2	309.33 ± 18.55	4.35 ± 0.79	2	163.53 ± 12.26	40.88 ± 3.57	2	318.31 ± 17.84	79.57 ± 7.46
*Eucephalobus*	2	43.13 ± 4.78	0.61 ± 0.11	2	70.56 ± 5.82	17.64 ± 2.23	2	218.20 ± 13.23	54.55 ± 6.12
*Rhabdolaimus $*	1	459.96 ± 21.57	6.47 ± 0.87	0	0.00 ± 0.00	0.00 ± 0.00	1	189.31 ± 11.21	47.32 ± 5.86
*Diplogaster*	1	406.23 ± 20.62	5.71 ± 0.66	1	124.96 ± 9.48	31.24 ± 3.15	1	273.79 ± 12.47	68.44 ± 5.98
*Diploscapter*	1	358.84 ± 19.43	5.05 ± 0.61	1	91.35 ± 6.09	22.84 ± 3.10	1	148.10 ± 10.33	37.02 ± 3.51
*Rhabditis*	1	242.28 ± 14.74	3.41 ± 0.43	1	110.82 ± 7.87	27.70 ± 3.79	1	215.97 ± 12.82	53.99 ± 4.76
*Teratorhabditis $*	1	373.49 ± 18.55	5.25 ± 0.72	0	0.00 ± 0.00	0.00 ± 0.00	1	133.21 ± 9.87	33.30 ± 3.43
*Mesorhabditis*	1	320.89 ± 16.91	4.51 ± 0.58	1	146.31 ± 11.23	36.58 ± 4.13	1	242.04 ± 13.42	60.51 ± 5.88
Cuticularia *	2	101.60 ± 6.68	1.43 ± 0.41	2	115.37 ± 6.89	28.84 ± 3.88	2	283.40 ± 15.21	70.84 ± 6.11
*Pelodera $*	1	253.99 ± 15.87	3.57 ± 0.59	0	0.00 ± 0.00	0.00 ± 0.00	1	193.18 ± 10.12	48.29 ± 4.57
*Protorhabditis*	1	14.94 ± 1.76	0.21 ± 0.08	1	79.55 ± 6.62	19.89 ± 2.58	1	288.86 ± 16.11	72.21 ± 6.74
**Omnivores**									
*Eudorylaimus*	4	292.84 ± 14.59	4.12 ± 0.57	4	170.41 ± 8.75	42.60 ± 4.54	4	115.45 ± 8.91	28.86 ± 3.45
Dorylaimus *	4	324.42 ± 18.52	4.56 ± 0.62	4	196.01 ± 8.87	49.00 ± 4.67	4	171.18 ± 12.13	42.79 ± 4.41
*Mesodorylaimus*	4	219.74 ± 12.23	3.09 ± 0.57	4	225.50 ± 12.26	56.37 ± 5.11	0	0.00 ± 0.00	0.00 ± 0.00
*Discolaimius*	5	181.83 ± 8.79	2.56 ± 0.36	5	132.07 ± 6.86	33.02 ± 3.24	5	81.06 ± 8.46	20.26 ± 2.78
**Predatory**									
*Mononchus*	1	253.99 ± 13.37	3.57 ± 0.60	1	64.92 ± 4.67	16.23 ± 2.28	1	97.69 ± 9.15	24.42 ± 2.89
*Mylonchulus $*	0	0.00 ± 0.00	0.00 ± 0.00	0	0.00 ± 0.00	0.00 ± 0.00	5	67.55 ± 7.13	16.89 ± 1.46
*Prionchulus*	4	171.29 ± 8.11	2.41 ± 0.29	4	25.25 ± 2.64	6.31 ± 1.04	4	46.99 ± 5.45	11.75 ± 1.31
*Clarkus*	1	11.95 ± 1.67	2.99 ± 0.47	1	30.81 ± 2.80	7.70 ± 0.95	0	0.00 ± 0.00	0.00 ± 0.00
*Miconchulus*	4	173.23 ± 7.88	2.44 ± 0.44	4	11.36 ± 1.34	2.84 ± 0.46	0	0.00 ± 0.00	0.00 ± 0.00
**Fungivores**									
*Dorylaimellus*	4	214.11 ± 11.49	3.01 ± 0.48	4	192.80 ± 9.86	48.20 ± 5.32	4	203.80 ± 12.25	50.95 ± 4.94
Dorylaimoides *	4	324.34 ± 18.33	4.56 ± 0.65	4	242.22 ± 12.48	60.56 ± 5.46	4	330.38 ± 14.64	82.59 ± 7.47
*Tylencholaimus*	1	38.03 ± 2.66	0.53 ± 0.13	1	139.57 ± 7.76	34.89 ± 3.43	1	283.40 ± 13.77	70.84 ± 6.88
*Aphelenchus*	0	0.00 ± 0.00	0.00 ± 0.00	2	231.86 ± 12.58	57.97 ± 4.89	2	300.75 ± 13.86	75.18 ± 7.14
*Aphelenchoides*	2	126.95 ± 5.87	1.78 ± 0.22	2	206.88 ± 11.59	51.72 ± 4.26	2	227.90 ± 12.57	56.97 ± 5.68

* Data represent the mean value ± standard error. The nematode genera with higher persistent prominence values are denoted with an asterisk (*) and lost persistence during the summer season is denoted with a dollar sign ($).

**Table 3:** The absolute and relative frequency, mean and relative density and prominence value and relative prominence value of plant-parasitic nematode genera found in rice root samples.

Nematode Genera	Absolute Frequency	Relative Frequency	Mean Density	Relative Density	PV	RPV
Hirschmanniella *	52.50 ± 8.22	4.87 ± 0.64	3.73 ± 0.23	188.21 ± 12.24	415.34 ± 34.54	31.60 ± 7.24
Meloidogyne *	48.00 ± 6.43	4.41 ± 0.51	3.52 ± 0.31	159.46 ± 10.37	334.87 ± 31.46	25.47 ± 4.35
*Paratylenchus*	27.50 ± 3.56	2.55 ± 0.23	2.17 ± 0.24	97.22 ± 8.45	155.25 ± 21.34	11.81 ± 2.78
*Xiphinema*	6.50 ± 0.68	0.58 ± 0.11	0.70 ± 0.12	41.43 ± 3.58	31.55 ± 6.65	2.40 ± 0.25
*Globodera*	11.00 ± 1.21	0.98 ± 0.48	1.16 ± 0.14	67.68 ± 5.42	70.00 ± 14.67	5.10 ± 0.55
Heterodera *	38.50 ± 4.50	3.21 ± 0.54	3.31 ± 0.32	147.96 ± 11.25	265.09 ± 30.22	20.16 ± 3.15
*Tylenchorhynchus*	6.50 ± 0.72	0.58 ± 0.13	1.27 ± 0.15	59.76 ± 4.13	45.51 ± 9.23	3.46 ± 0.80

Data are represented as mean value ± standard error and the nematode genera with higher frequency, density, and prominence values are denoted by asterisks (*).

**Table 4:** Community and diversity indices of soil nematodes of the rice fields during the spring, summer, and winter seasons of the year.

Indices	Spring Season	Summer Season	Winter Season
Shannon–Wiener -ndex (H’)	3.47 ± 0.55 ^b^	3.60 ± 0.57 ^a^	3.40 ± 0.51 ^c^
Pielou’s evenness (J′)	0.002 ± 0.001 ^c^	1.00 ± 0.08 ^a^	0.90 ± 0.08 ^b^
Simpson index (D)	0.97 ± 0.06 ^a^	0.002 ± 0.001 ^b^	0.002 ± 0.001 ^b^
Margalef Index (MgI)	5.80 ± 0.78 ^b^	6.21 ± 0.72 ^a^	5.60 ± 0.65 ^c^
Wasilewska index (WI)	1.15 ± 0.09 ^b^	0.82 ± 0.06 ^c^	1.50 ± 0.11 ^a^
Channel index (CI)	3.03 ± 0.42 ^c^	9.10 ± 1.03 ^a^	5.50 ± 0.58 ^b^
Food web complexity (FWC)	0.60 ± 0.04 ^a^	0.50 ± 0.03 ^b^	0.50 ± 0.04 ^b^
Maturity index (MI)	2.40 ± 0.22 ^a^	2.30 ± 0.46 ^b^	2.20 ± 0.24 ^c^
Plant-parasitic index (PPI)	2.78 ± 0.27 ^b^	2.97 ± 0.21 ^a^	2.47 ± 0.25 ^c^

Data are represented as mean value ± standard error (n = 72) and different letters after data show the significance at *p* ≤ 0.05 during the different seasons.

## Data Availability

The data presented in this study are available in the Supplementary Materials.

## Supplementary Materials

The following supporting information can be downloaded at: https://www.mdpi.com/article/10.3390/biology11111572/s1, Table S1: Datasets of frequency and density and their relative data of nematode genera during the three seasons in the soil of the rice fields.
