# Supplementary material for: Year-Long Assessment of Soil Nematode Diversity and Root Inhibition-Indicator Nematode Genera in Rice Fields
Source: Biology (Basel). 2022 Oct 26;11(11):1572. doi: 10.3390/biology11111572 (PMC9687600; doi:10.3390/biology11111572)
Supplement: Supplementary file 1 [file biology-11-01572-s001.zip › biology-1952229-supplementary.pdf]

Year-Long Assessment of Soil Nematode Diversity and Root Inhibition-Indicator Nematode Genera in Rice Fields

Rawhat Un Nisa [1], Anees Un Nisa [2], Ali Ahmed Hroobi [3], Ali Asghar Shah [1] and Aadil Yousuf Tantray [4,5,*]

[1] Nematode Biodiversity & Genomics Research Lab, BGSB University, Rajouri, 185234, India
[2] Mycology & Plant Pathology Lab, Department of Botany, University of Kashmir, Srinagar, 190006, India
[3] Department of Biology, College of Sciences in Almandaq, Al-Baha University, Al-Baha, 65779-7738, Saudi Arabia
[4] Department of Botany, Aligarh Muslim University, Aligarh, 202002, India
[5] School of Biological Sciences, University of Aberdeen, Aberdeen, AB243UU, UK
*Correspondence: a.y.tantray@gmail.com; Tel.: +91 8493082705

Supplementary Table S1: Datasets of frequency, density, and their relative data of nematode genera during the three seasons in the soil of the rice fields.

A. **Before plantation (season I)**

Nematode genera	Frequency	Relative frequency	Absolute frequency	Density	Relative density	Mean density	Nematode no.	c-p values
**Plant-parasitic**								
Psilenchus	10.00	3.70	25.00	80.00	130.93	2.00	12.00	2
Radopholus	6.00	2.22	15.00	65.00	106.38	1.63	8.00	2
*Longidorus**	9.00	3.33	22.50	73.00	119.48	1.83	13.00	5
Rotylenchus	0.00	0.00	0.00	0.00	0.00	0.00	0.00	0
Criconemoides$	2.00	0.74	5.00	22.00	36.01	0.55	5.00	3
Ditylenchus	5.00	1.85	12.50	46.00	75.29	1.15	8.00	2
*Meloidogyne**	7.00	2.59	17.50	53.00	86.74	1.33	8.00	3
Helicotylenchus	5.00	1.85	12.50	45.00	73.65	1.13	6.00	3
*Heterodera**	8.00	2.96	20.00	74.00	121.11	1.85	13.00	3
*Hirschmanniella**	5.00	1.85	12.50	50.00	81.83	1.25	7.00	3
Hoplolaimus	0.00	0.00	0.00	0.00	0.00	0.00	0.00	0
Globodera	3.00	1.11	7.50	32.00	52.37	0.80	5.00	3

*Tylenchus**	9.00	3.33	22.50	85.00	139.12	2.13	15.00	2
Paratylenchus	0.00	0.00	0.00	0.00	0.00	0.00	0.00	0
Pratylenchus	4.00	1.48	10.00	48.00	78.56	1.20	6.00	3
Hexatylenchus	0.00	0.00	0.00	0.00	0.00	0.00	0.00	0
Tylenchorhynchus	6.00	2.22	15.00	69.00	112.93	1.73	8.00	2
Xiphinema	0.00	0.00	0.00	0.00	0.00	0.00	0.00	0
**Bacteriovores**								
Acrobelus	0.00	0.00	0.00	0.00	0.00	0.00	0.00	0
*Cephalobus**	1.00	0.37	2.50	15.00	24.55	0.38	4.00	2
Eucephalobus	14.00	5.19	35.00	83.00	135.84	2.08	18.00	2
Rhabdolaimus$	3.00	1.11	7.50	25.00	40.92	0.63	7.00	1
Diplogaster	17.00	6.30	42.50	112.00	183.31	2.80	25.00	1
Diploscapter	14.00	5.19	35.00	109.00	178.40	2.73	21.00	1
Rhabditis	12.00	4.44	30.00	104.00	170.21	2.60	16.00	1
Teratorhabditis$	8.00	2.96	20.00	86.00	140.75	2.15	13.00	1
Mesorhabditis	13.00	4.81	32.50	104.00	170.21	2.60	19.00	1
*Cuticularia**	12.00	4.44	30.00	93.00	152.21	2.33	16.00	2
Pelodera$	4.00	1.48	10.00	51.00	83.47	1.28	6.00	1

Protorhabditis	9.00	3.33	22.50	85.00	139.12	2.13	11.00	1
**Omnivores**								
Eudorylaimus	9.00	3.33	22.50	98.00	160.39	2.45	11.00	4
*Dorylaimus**	10.00	3.70	25.00	103.00	168.58	2.58	18.00	4
Mesodorylaimus	8.00	2.96	20.00	78.00	127.66	1.95	10.00	4
Discolaimius	7.00	2.59	17.50	69.00	112.93	1.73	9.00	5
**Predatory**								
Mononchus	9.00	3.33	22.50	85.00	139.12	2.13	13.00	1
Mylonchulus$	0.00	0.00	0.00	0.00	0.00	0.00	0.00	0
Prionchulus	7.00	2.59	17.50	65.00	106.38	1.63	9.00	4
Clarkus	1.00	0.37	2.50	12.00	19.64	0.30	4.00	1
Miconchulus	6.00	2.22	15.00	71.00	116.20	1.78	8.00	4
**Fungivores**								
Dorylaimellus	8.00	2.96	20.00	76.00	124.39	1.90	14.00	4
*Dorylaimoides**	12.00	4.44	30.00	94.00	153.85	2.35	15.00	4
Tylencholaimus	2.00	0.74	5.00	27.00	44.19	0.68	5.00	1
Aphelenchus	0.00	0.00	0.00	0.00	0.00	0.00	0.00	0
Aphelenchoides	5.00	1.85	12.50	57.00	93.29	1.43	9.00	2

Data is represented in mean values of each diversity trait. The nematode genera which were with higher density and frequency values are denoted with asterisk (*) and which lost persistence at flood irrigation are denoted with dollar ($).

B. During plantation (season II)

Nematode genera	Frequency	Relative frequency	Absolute frequency	Density	Relative density	Mean density	Nematode no.	c-p values
**Plant-parasitic**								
Psilenchus	16.00	3.71	40.00	110.00	131.03	2.75	12.00	2
Radopholus	13.00	3.02	32.50	100.00	119.12	2.50	13.00	2
*Longidorus**	18.00	4.18	45.00	120.00	142.94	3.00	16.00	5
Rotylenchus	9.00	2.09	22.50	88.00	104.82	2.20	12.00	1
Criconemoides$	0.00	0.00	0.00	0.00	0.00	0.00	0.00	0
Ditylenchus	19.00	4.41	47.50	130.00	154.85	3.25	20.00	2
*Meloidogyne**	20.00	4.64	50.00	137.00	163.19	3.43	14.00	3
Helicotylenchus	14.00	3.25	35.00	95.00	113.16	2.38	9.00	3
*Heterodera**	16.00	3.71	40.00	102.00	121.50	2.55	14.00	3
*Hirschmanniella**	23.00	5.34	57.50	117.00	139.37	2.93	21.00	3
Hoplolaimus	12.00	2.78	30.00	98.00	116.74	2.45	16.00	1
Globodera	11.00	2.55	27.50	88.00	104.82	2.20	8.00	3
*Tylenchus**	20.00	4.64	50.00	96.00	114.35	2.40	19.00	2

Paratylenchus	21.00	4.87	52.50	123.00	146.52	3.08	17.00	1
Pratylenchus	17.00	3.94	42.50	104.00	123.88	2.60	16.00	3
Hexatylenchus	9.00	2.09	22.50	83.00	98.87	2.08	11.00	2
Tylenchorhynchus	4.00	0.93	10.00	46.00	54.79	1.15	5.00	2
Xiphinema	3.00	0.70	7.50	28.00	33.35	0.70	8.00	1
**Bacteriovores**								
Acrobelus	11.00	2.55	27.50	100.00	119.12	2.50	8.00	2
*Cephalobus**	9.00	2.09	22.50	95.00	113.16	2.38	7.00	2
Eucephalobus	5.00	1.16	12.50	55.00	65.52	1.38	9.00	2
Rhabdolaimus$	0.00	0.00	0.00	0.00	0.00	0.00	0.00	0
Diplogaster	8.00	1.86	20.00	77.00	91.72	1.93	11.00	1
Diploscapter	6.00	1.39	15.00	65.00	77.43	1.63	14.00	1
Rhabditis	7.00	1.62	17.50	73.00	86.96	1.83	9.00	1
Teratorhabditis$	0.00	0.00	0.00	0.00	0.00	0.00	0.00	0
Mesorhabditis	9.00	2.09	22.50	85.00	101.25	2.13	16.00	1
*Cuticularia**	7.00	1.62	17.50	76.00	90.53	1.90	19.00	2
Pelodera$	0.00	0.00	0.00	0.00	0.00	0.00	0.00	0
Protorhabditis	5.00	1.16	12.50	62.00	73.85	1.55	13.00	1

**Omnivores**								
Eudorylaimus	9.00	2.09	22.50	99.00	117.93	2.48	9.00	4
*Dorylaimus**	11.00	2.55	27.50	103.00	122.69	2.58	17.00	4
Mesodorylaimus	13.00	3.02	32.50	109.00	129.84	2.73	13.00	4
Discolaimius	7.00	1.62	17.50	87.00	103.63	2.18	8.00	5
**Predatory**								
Mononchus	8.00	1.86	20.00	40.00	47.65	1.00	7.00	1
Mylonchulus$	0.00	0.00	0.00	0.00	0.00	0.00	0.00	0
Prionchulus	4.00	0.93	10.00	22.00	26.21	0.55	3.00	4
Clarkus	3.00	0.70	7.50	31.00	36.93	0.78	4.00	1
Miconchulus	2.00	0.46	5.00	14.00	16.68	0.35	5.00	4
**Fungivores**								
Dorylaimellus	12.00	2.78	30.00	97.00	115.54	2.43	12.00	4
*Dorylaimoides**	15.00	3.48	37.50	109.00	129.84	2.73	9.00	4
Tylencholaimus	8.00	1.86	20.00	86.00	102.44	2.15	8.00	1
Aphelenchus	14.00	3.25	35.00	108.00	128.65	2.70	10.00	2
Aphelenchoides	13.00	3.02	32.50	100.00	119.12	2.50	11.00	2

Data is represented in mean values of each diversity trait. The nematode genera which were with higher density and frequency values are denoted with asterisk (*) and which lost persistence at flood irrigation are denoted with dollar ($).

C. **After harvesting (season III)**

Nematode genera	Frequency	Relative frequency	Absolute frequency	Density	Relative density	Mean density	Nematode no.	c-p values
**Plant-parasitic**								
Psilenchus	0.00	0.00	0.00	0.00	0.00	0.00	0.00	0
Radopholus	0.00	0.00	0.00	0.00	0.00	0.00	0.00	0
*Longidorus**	12.00	3.11	30.00	104.00	137.16	2.60	9.00	5
Rotylenchus	9.00	2.33	22.50	83.00	109.47	2.08	10.00	1
Criconemoides$	0.00	0.00	0.00	0.00	0.00	0.00	0.00	0
Ditylenchus	9.00	2.33	22.50	78.00	102.88	1.95	13.00	2
*Meloidogyne**	12.00	3.11	30.00	89.00	117.38	2.23	7.00	3
Helicotylenchus	0.00	0.00	0.00	0.00	0.00	0.00	0.00	0
*Heterodera**	14.00	3.63	35.00	107.00	141.12	2.68	9.00	3
*Hirschmanniella**	17.00	4.40	42.50	113.00	149.04	2.83	10.00	3
Hoplolaimus	0.00	0.00	0.00	0.00	0.00	0.00	0.00	0
Globodera	9.00	2.33	22.50	75.00	98.92	1.88	6.00	3
*Tylenchus**	16.00	4.15	40.00	100.00	131.89	2.50	11.00	2
Paratylenchus	15.00	3.89	37.50	88.00	116.06	2.20	10.00	1
Pratylenchus	13.00	3.37	32.50	78.00	102.88	1.95	8.00	3

Hexatylenchus	7.00	1.81	17.50	55.00	72.54	1.38	7.00	2
Tylenchorhynchus	0.00	0.00	0.00	0.00	0.00	0.00	0.00	0
Xiphinema	0.00	0.00	0.00	0.00	0.00	0.00	0.00	0
**Bacteriovores**								
Acrobelus	16.00	4.15	40.00	112.00	147.72	2.80	5.00	2
*Cephalobus**	17.00	4.40	42.50	115.00	151.68	2.88	10.00	2
Eucephalobus	11.00	2.85	27.50	98.00	129.25	2.45	9.00	2
Rhabdolaimus $	9.00	2.33	22.50	94.00	123.98	2.35	11.00	1
Diplogaster	14.00	3.63	35.00	109.00	143.76	2.73	14.00	1
Diploscapter	8.00	2.07	20.00	78.00	102.88	1.95	10.00	1
Rhabditis	11.00	2.85	27.50	97.00	127.93	2.43	13.00	1
Teratorhabditis$	7.00	1.81	17.50	75.00	98.92	1.88	15.00	1
Mesorhabditis	13.00	3.37	32.50	100.00	131.89	2.50	9.00	1
*Cuticularia**	15.00	3.89	37.50	109.00	143.76	2.73	17.00	2
Pelodera$	10.00	2.59	25.00	91.00	120.02	2.28	7.00	1
Protorhabditis	14.00	3.63	35.00	115.00	151.68	2.88	18.00	1
**Omnivores**								
Eudorylaimus	7.00	1.81	17.50	65.00	85.73	1.63	9.00	4

*Dorylaimus**	9.00	2.33	22.50	85.00	112.11	2.13	10.00	4
Mesodorylaimus	0.00	0.00	0.00	0.00	0.00	0.00	0.00	0
Discolaimius	5.00	1.30	12.50	54.00	71.22	1.35	8.00	5
**Predatory**								
Mononchus	7.00	1.81	17.50	55.00	72.54	1.38	8.00	1
Mylonchulus$	5.00	1.30	12.50	45.00	59.35	1.13	9.00	5
Prionchulus	4.00	1.04	10.00	35.00	46.16	0.88	8.00	4
Clarkus	0.00	0.00	0.00	0.00	0.00	0.00	0.00	0
Miconchulus	0.00	0.00	0.00	0.00	0.00	0.00	0.00	0
**Fungivores**								
Dorylaimellus	10.00	2.59	25.00	96.00	126.62	2.40	15.00	4
*Dorylaimoides**	18.00	4.66	45.00	116.00	152.99	2.90	16.00	4
Tylencholaimus	15.00	3.89	37.50	109.00	143.76	2.73	11.00	1
Aphelenchus	16.00	4.15	40.00	112.00	147.72	2.80	15.00	2
Aphelenchoides	12.00	3.11	30.00	98.00	129.25	2.45	14.00	2

Data is represented in mean values of each diversity trait. The nematode genera which were with higher density and frequency values are denoted with asterisk (*) and which lost persistence at flood irrigation are denoted with dollar ($).
